# The impact of preoperative fasting time on patients undergoing partial nephrectomy and analysis of risk factors for postoperative hemorrhage

**DOI:** 10.3389/fsurg.2024.1474910

**Published:** 2024-10-03

**Authors:** Chunji Wang, Jiazhao Cui, Zihui Gao

**Affiliations:** Department of Urology, Peking University First Hospital - MiYun Hospital, Beijing, China

**Keywords:** partial nephrectomy, fasting time, hemorrhage, risk factors, type of surgery

## Abstract

**Objective:**

This study investigates the impact of preoperative fasting time on patients undergoing partial nephrectomy and analyzes the risk factors for postoperative hemorrhage to provide clinical reference for physicians treating patients undergoing partial nephrectomy.

**Methods:**

A retrospective analysis was conducted on 74 patients who underwent partial nephrectomy for renal tumors between January 2022 and March 2024. Baseline and perioperative data were collected. The effects of long-term and short-term preoperative fasting on patients undergoing partial nephrectomy were compared. Additionally, univariate and multivariate logistic regression analyses were performed to identify risk factors for hemorrhagic complications following partial nephrectomy.

**Results:**

Among the patients in this study, 26 (35.14%) underwent short-term preoperative fasting, while 48 (64.86%) underwent long-term preoperative fasting. The hemoglobin difference in the short-term fasting group was 21.08 ± 12.44 ml, compared to 13.65 ± 11.69 ml in the long-term fasting group, showing a statistically significant difference (*p* = 0.020). Differences in serum calcium (*p* = 0.003), serum magnesium (*p* = 0.031), and serum phosphorus (*p* = 0.001) between the short-term and long-term fasting groups were also statistically significant. Univariate and multivariate regression analyses identified the type of surgery (*p* = 0.050) and preoperative fasting time (*p* < 0.001) as independent risk factors for postoperative hemorrhage following partial nephrectomy.

**Conclusion:**

Patients undergoing partial nephrectomy with short-term preoperative fasting experience a more significant decrease in hemoglobin compared to those with long-term fasting. The type of surgery and preoperative fasting time are independent risk factors for postoperative hemorrhage in patients undergoing partial nephrectomy.

## 1 Introduction

In recent years, the incidence of renal cancer diagnoses has been steadily increasing. According to the American Cancer Society, there were 73,750 new cases of renal cancer diagnosed in 2020 (1, 2). Currently, the surgical treatment for renal cancer is primarily divided into partial nephrectomy and radical nephrectomy. When technically feasible, nephron-sparing surgery is recommended as the preferred surgical treatment for renal tumors. As a result, partial nephrectomy has become the gold standard for surgical treatment of T1 renal cell carcinoma (3–6).

Long periods of preoperative fasting can lead to dehydration, electrolyte imbalance, and reduced blood volume, which in turn can weaken the immune system and increase the risk of postoperative infections (7–9). It may also result in gastrointestinal discomfort during postoperative recovery and adversely affect the recovery process. This study aims to investigate the impact of preoperative fasting time on patients undergoing partial nephrectomy.

Hemorrhage is one of the common complications following partial nephrectomy. It can not only affect the recovery of patients but, in severe cases, can be life-threatening (10). Although hemostatic drugs can control bleeding to some extent, some severe cases require vascular embolization or even reoperation (11, 12). Therefore, the factors contributing to postoperative hemorrhage following partial nephrectomy warrant attention. This study analyzes the risk factors for hemorrhage following partial nephrectomy to provide clinical reference for physicians in their treatment approaches.

## 2 Methods

A total of 74 renal cancer patients underwent partial nephrectomy at Peking University First Hospital-Miyun hospital between January 2022 and March 2024. Among these patients, 26 had a short-term fasting time, while 48 had a long-term preoperative fasting time. Baseline and perioperative data were collected for all patients. Baseline data included gender, age, body mass index (BMI), presence of hypertension, presence of diabetes, and presence of coronary artery disease (CHD). Perioperative data included tumor size, type of surgery, heart rate at admission, systolic blood pressure at admission, diastolic blood pressure at admission, preoperative creatinine, preoperative blood sodium, preoperative blood potassium, preoperative blood magnesium, preoperative blood phosphorus, preoperative blood calcium, preoperative hemoglobin, intraoperative heart rate, intraoperative systolic blood pressure, intraoperative diastolic blood pressure, postoperative heart rate, postoperative systolic blood pressure, postoperative diastolic blood pressure, surgery duration, intraoperative blood loss, postoperative creatinine, postoperative blood sodium, postoperative blood potassium, postoperative blood magnesium, postoperative blood phosphorus, postoperative blood calcium, and postoperative hemoglobin.

In this study, short-term preoperative fasting was defined as a fasting time of 12 h or less, while long-term preoperative fasting was defined as more than 12 h. The primary endpoint for the analysis of risk factors was postoperative hemorrhage. Postoperative hemorrhage was defined as a decrease in hemoglobin of 20 g/L or more between preoperative levels and the first postoperative day. A decrease of less than 20 g/L was not considered a postoperative hemorrhage complication. The decrease in hemoglobin (g/L) was calculated as preoperative hemoglobin level minus the hemoglobin level on the first postoperative day.

Inclusion criteria: a. Patients aged 18–75 years with T1 tumors and a R.E.N.A.L. nephrometry score of ≤9; b. Complete baseline and perioperative data.

Exclusion criteria: a. Patients with uncontrolled severe disease or acute infection; b. Pregnant or lactating women.

This study was conducted in accordance with the principles of the Declaration of Helsinki (2013 revision) and was approved by the Ethics Committee of Peking University First Hospital-Miyun hospital. The retrospective analysis of this study was exempted from individual consent.

### 2.1 Surgical technique

The patient is placed in a supine position, and standard disinfection and draping are performed. After establishing pneumoperitoneum, trocars are inserted. Using a laparoscope and electrocautery or scissors, the surrounding fat and Gerota's fascia are dissected to expose the kidney and tumor. The renal artery is temporarily clamped using vascular clamps or other devices to reduce intraoperative bleeding. This step must be performed quickly and accurately to minimize renal ischemia time. The tumor and a small margin of surrounding healthy renal tissue are excised using laparoscopic surgical instruments, ensuring negative margins. The renal incision is repaired using absorbable sutures or Hem-o-lok clips to restore the integrity of the kidney. Hemostatic agents or fibrin glue may be used to assist in hemostasis. The renal artery clamp is then removed to restore renal blood flow. The surgical field is checked for bleeding or other complications, and once confirmed to be clear, the laparoscopic instruments are removed and the small abdominal incisions are closed.

## 3 Statistical analysis

Statistical analysis was performed using SPSS 22.0 (IBM Corp., Armonk, NY, USA). Quantitative variables included age, BMI, tumor size, heart rate at admission, systolic blood pressure at admission, diastolic blood pressure at admission, preoperative creatinine, preoperative blood sodium, preoperative blood potassium, preoperative blood magnesium, preoperative blood phosphorus, preoperative blood calcium, preoperative hemoglobin, intraoperative heart rate, intraoperative systolic blood pressure, intraoperative diastolic blood pressure, postoperative heart rate, postoperative systolic blood pressure, postoperative diastolic blood pressure, surgery duration, intraoperative blood loss, postoperative creatinine, postoperative blood sodium, postoperative blood potassium, postoperative blood magnesium, postoperative blood phosphorus, postoperative blood calcium, and postoperative hemoglobin. Qualitative variables included gender, presence of hypertension, presence of diabetes, presence of CHD, and type of surgery. Normally distributed quantitative data were expressed as mean ± standard deviation, while non-normally distributed data were described using median (range). For continuous variables, normally distributed variables were analyzed using the *t*-test, while the Mann-Whitney *U*-test was used for non-normally distributed variables. Categorical variables were analyzed using Fisher's exact test. Univariate and multivariate regression analyses were conducted to determine independent risk factors for postoperative hemorrhage following partial nephrectomy. A *p*-value of <0.05 was considered statistically significant.

## 4 Results

### 4.1 Patient baseline data

The baseline data of the patients are shown in Table 1. A total of 74 patients who underwent partial nephrectomy were included in this study, with an average age of 52.45 ± 12.75 years. The average tumor size was 3.42 ± 1.70 cm. There were 26 (35.14%) patients in the short-term preoperative fasting group and 48 (64.86%) patients in the long-term preoperative fasting group. Among them, 45 patients experienced postoperative complications, with 12 patients having two complications. No postoperative complications of grade III or higher were observed. The incidence of grade I complications was 39.19% (29/74), and the incidence of grade II complications was 36.49% (27/74).

**Table 1:** Basic characteristics of the patients.

Variable	Mean (SD) or *n*/*N*
Patients	74
Mean age (years)	52.45 ± 12.75
BMI (kg/m2)	25.52 ± 3.45
Gender, *n* (%)	
Male	40 (54.05)
Female	34 (45.95)
Hypertension, *n* (%)	
Yes	27 (36.49)
No	47 (63.51)
Diabetes mellitus, *n* (%)	
Yes	17 (22.97)
No	57 (77.03)
CHD, *n* (%)	
Yes	4 (5.41)
No	70 (94.59)
Type of Surgery, *n* (%)	
Laparoscopy	55 (74.32)
Robot-assisted	19 (25.68)
Tumor size (cm)	3.42 ± 1.70
RENAL score	5.01 ± 1.53
Warm ischemia time (minutes)	21.31 ± 3.69
Admission heart rate (beats per minute)	77.81 ± 8.50
Admission systolic blood Pressure (mmHg)	133.54 ± 14.76
Admission diastolic blood pressure (mmHg)	84.14 ± 11.11
Preoperative creatinine (μmol/L)	66.11 ± 19.11
Preoperative blood sodium (mmol/L)	139.90 ± 2.01
Preoperative blood potassium (mmol/L)	3.97 ± 0.33
Preoperative blood calcium (mmol/L)	2.29 ± 0.10
Preoperative blood phosphate (mmol/L)	1.18 ± 0.18
Preoperative blood magnesium (mmol/L)	0.86 ± 0.06
Preoperative hemoglobin (g/L)	139.49 ± 18.59
Intraoperative heart rate (beats per minute)	60.73 ± 10.10
Intraoperative systolic blood pressure (mmHg)	115.04 ± 17.21
Intraoperative diastolic blood pressure (mmHg)	62.31 ± 10.32
Postoperative heart rate (beats per minute)	75.15 ± 10.72
Postoperative systolic blood pressure (mmHg)	134.97 ± 16.17
Postoperative diastolic blood pressure (mmHg)	80.62 ± 10.54
Surgery duration (min)	163.51 ± 58.08
Intraoperative blood loss (ml)	66.81 ± 102.92
Postoperative hospital stay (days)	6.62 ± 2.30
Postoperative creatinine (μmol/L)	74.29 ± 21.03
Postoperative blood sodium (mmol/L)	139.26 ± 2.01
Postoperative blood potassium (mmol/L)	3.73 ± 0.45
Postoperative blood calcium (mmol/L)	2.12 ± 0.10
Postoperative blood phosphate (mmol/L)	1.03 ± 0.35
Postoperative blood magnesium (mmol/L)	0.80 ± 0.06
Postoperative hemoglobin (g/L)	120.48 ± 19.24
Fasting time, *n* (%)	
≤12h	26 (35.14）
>12h	48 (64.86）
Clavien-Dindo grade, *n* (%)	
Grade I	29 (39.19)
Bleeding	20 (27.03)
Nausea and vomiting	9 (12.16)
Grade II	27 (36.49)
Bleeding (iron supplementation)	14 (18.92)
Infection (antibiotics)	13 (17.57)
Grade III-V	0 (0.00)

BMI, body mass index; CHD, coronary heart disease.

### 4.2 Comparison between short-term and long-term preoperative fasting in partial nephrectomy

The clinical data of patients in the short-term and long-term preoperative fasting groups are shown in Table 2. The hemoglobin difference in the short-term preoperative fasting group was 21.08 ± 12.44 ml, while in the long-term preoperative fasting group it was 13.65 ± 11.69 ml, showing a statistically significant difference between the two groups (*p* = 0.020). The calcium difference in the short-term preoperative fasting group was 0.96 ± 1.01 mmol/L, compared to 0.08 ± 0.11 mmol/L in the long-term preoperative fasting group, also showing a statistically significant difference (*p* = 0.003). The phosphorus difference was 0.54 ± 0.52 mmol/L in the short-term preoperative fasting group and 0.09 ± 0.33 mmol/L in the long-term preoperative fasting group, with a statistically significant difference (*p* = 0.001). The magnesium difference was 0.35 ± 0.40 mmol/L in the short-term preoperative fasting group and 0.02 ± 0.05 mmol/L in the long-term preoperative fasting group, again showing a statistically significant difference (*p* = 0.031).

**Table 2:** Comparing the difference in prognosis between short-term fasting group and long-term fasting group.

Variable	Short-term fasting	Long-term fasting	*p* value
Patients	26	48	
Mean age (years)	55.08 ± 13.83	51.02 ± 11.89	0.196
BMI (kg/m2)	25.37 ± 3.21	25.61 ± 3.57	0.772
Gender, *n* (%)			0.15
Male	17 (65.38)	23 (47.92)	
Female	9 (34.62)	25 (52.08)	
Hypertension, *n* (%)			0.795
Yes	10 (38.46)	17 (35.42)	
No	16 (61.54)	31 (64.58)	
Diabetes mellitus, *n* (%)			0.988
Yes	6 (23.08)	11 (22.92)	
No	20 (76.92)	37 (77.08)	
CHD, *n* (%)			0.662
Yes	1 (3.85)	3 (6.25)	
No	25 (96.15)	45 (93.75)	
Type of surgery, *n* (%)			0.717
Laparoscopy	18 (69.23)	37 (77.08)	
Robot-assisted	8 (30.77)	11 (22.92)	
Tumor size (cm)	3.73 ± 1.81	3.25 ± 1.64	0.254
RENAL score	5.04 ± 1.73	5.00 ± 1.39	0.363
Warm ischemia time (minutes)	20.77 ± 3.63	21.60 ± 3.66	0.920
Heart rate difference (beats per minute)	0.12 ± 13.10	4.04 ± 12.44	0.215
Systolic blood pressure difference (mmHg)	−2.81 ± 20.42	−0.69 ± 21.02	0.676
Diastolic blood pressure difference (mmHg)	2.46 ± 12.04	4.08 ± 12.93	0.599
Creatinine difference (μmol/L)	−8.38 ± 14.28	−0.15 ± 26.55	0.162
Blood sodium difference (mmol/L)	10.95 ± 37.09	0.40 ± 1.94	0.365
Blood potassium difference (mmol/L)	0.47 ± 1.17	0.20 ± 0.47	0.205
Blood calcium difference (mmol/L)	0.96 ± 1.01	0.08 ± 0.11	0.003
Blood phosphate difference (mmol/L)	0.54 ± 0.52	0.09 ± 0.33	0.001
Blood magnesium difference (mmol/L)	0.35 ± 0.40	0.02 ± 0.05	0.031
Hemoglobin difference (g/L)	21.08 ± 12.44	13.65 ± 11.69	0.020
Intraoperative heart rate (beats per minute)	59.5 ± 8.42	61.40 ± 10.85	0.444
Intraoperative systolic blood pressure (mmHg)	110.73 ± 18.47	117.38 ± 16.00	0.119
Intraoperative diastolic blood pressure (mmHg)	60.5 ± 12.00	63.29 ± 9.14	0.271
Surgery duration (min)	157.42 ± 46.70	165 ± 63.31	0.570
Intraoperative blood loss (ml)	62.88 ± 79.69	68.98 ± 113.70	0.832
Postoperative hospital stay (days)	6.81 ± 2.56	6.52 ± 2.14	0.610
Clavien-Dindo grade, *n* (%)			
Grade I	22 (84.62)	7 (14.58)	<0.001
Bleeding	17 (65.38)	3 (6.25)	
Nausea and vomiting	5 (19.23)	4 (8.33)	
Grade II	15 (57.69)	12 (25.00)	0.005
Bleeding (iron supplementation)	9 (34.62)	5 (10.42)	
Infection (antibiotics)	6 (23.08)	7 (14.58)	
Grade III-V	0 (0.00)	0 (0.00)	

### 4.3 Analysis of risk factors for hemorrhage after partial nephrectomy

The clinical data of the control group and the hemorrhage group are shown in Table 3. Postoperatively, there were 40 (54.05%) patients in the control group and 34 (45.95%) patients in the hemorrhage group. In the control group, 34 (85.00%) patients underwent laparoscopic partial nephrectomy and 6 (15.00%) underwent robot-assisted partial nephrectomy. In the hemorrhage group, 21 (61.76%) patients underwent laparoscopic partial nephrectomy and 13 (38.24%) underwent robot-assisted partial nephrectomy, showing a statistically significant difference between the two groups (*p* = 0.023). In the control group, 2 (5.00%) patients were in the short-term preoperative fasting group, and 38 (95.00%) were in the long-term preoperative fasting group. In the hemorrhage group, 24 (70.59%) patients were in the short-term preoperative fasting group, and 10 (29.41%) were in the long-term preoperative fasting group, also showing a statistically significant difference (*p* < 0.001).

**Table 3:** Comparing the difference in prognosis between control group and bleeding group.

Variable	Control group	Bleeding group	*p* value
Patients	40	34	
Mean age (years)	52.4 ± 11.81	52.5 ± 13.78	0.268
BMI (kg/m^2^)	25.80 ± 3.56	25.20 ± 3.28	0.989
Gender, *n* (%)			0.09
Male	18 (45)	22 (64.71)	
Female	22 (55)	12 (35.29)	
Hypertension, *n* (%)			0.773
Yes	14 (35)	13 (38.24)	
No	26 (65)	21 (61.76)	
Diabetes mellitus, *n* (%)			0.315
Yes	11 (27.5)	6 (17.65)	
No	29 (72.5)	28 (82.35)	
CHD, *n* (%)			0.387
Yes	3 (12.5)	1 (2.94)	
No	37 (87.5)	33 (97.06)	
Type of surgery, *n* (%)			0.023
Laparoscopy	34 (85)	21 (61.76)	
Robot-assisted	6 (15)	13 (38.24)	
Tumor size (cm)	3.24 ± 1.57	3.61 ± 1.80	0.052
RENAL score	4.90 ± 1.38	5.15 ± 1.74	0.498
Warm ischemia time (minutes)	21.60 ± 3.64	20.97 ± 3.90	0.475
Admission heart rate (beats per minute)	80.08 ± 7.51	75.15 ± 8.81	0.464
Admission systolic blood pressure (mmHg)	134.8 ± 15.34	132.06 ± 13.89	0.638
Admission diastolic blood pressure (mmHg)	85.55 ± 11.45	82.47 ± 10.46	0.938
Preoperative creatinine (μmol/L)	60.91 ± 14.94	72.42 ± 21.54	0.461
Preoperative blood sodium (mmol/L)	140.00 ± 2.00	139.71 ± 2.03	0.521
Preoperative blood potassium (mmol/L)	3.95 ± 0.34	3.98 ± 0.32	0.533
Preoperative blood calcium (mmol/L)	2.28 ± 0.10	2.30 ± 0.10	0.747
Preoperative blood phosphate (mmol/L)	1.14 ± 0.17	1.20 ± 0.18	0.771
Preoperative blood magnesium (mmol/L)	0.86 ± 0.06	0.86 ± 0.06	0.964
Preoperative hemoglobin (g/L)	136.82 ± 18.95	141.21 ± 18.21	0.728
Intraoperative heart rate (beats per minute)	61.95 ± 11.22	59.29 ± 8.26	0.24
Intraoperative systolic blood pressure (mmHg)	117.63 ± 16.34	112 ± 17.44	0.928
Intraoperative diastolic blood pressure (mmHg)	63.32 ± 9.07	61.12 ± 11.34	0.298
Postoperative heart rate (beats per minute)	75.23 ± 11.53	75.06 ± 9.55	0.17
Postoperative systolic blood pressure (mmHg)	134.33 ± 16.14	135.74 ± 15.94	0.758
Postoperative diastolic blood pressure (mmHg)	79.98 ± 10.39	81.38 ± 10.51	0.97
Surgery duration (min)	163.48 ± 61.36	161.68 ± 53.33	0.457
Intraoperative blood loss (ml)	73.3 ± 121.92	58.09 ± 70.87	0.217
Postoperative hospital stay (days)	6.45 ± 2.01	6.82 ± 2.55	0.346
Postoperative creatinine (μmol/L)	69.30 ± 20.10	79.58 ± 20.39	0.743
Postoperative blood sodium (mmol/L)	139.64 ± 2.03	139.41 ± 1.99	0.93
Postoperative blood potassium (mmol/L)	3.74 ± 0.42	3.81 ± 0.48	0.477
Postoperative blood calcium (mmol/L)	2.19 ± 0.13	2.15 ± 0.11	0.418
Postoperative blood phosphate (mmol/L)	1.08 ± 0.38	1.09 ± 0.17	0.179
Postoperative blood magnesium (mmol/L)	0.83 ± 0.07	0.83 ± 0.08	0.491
Fasting Time			<0.001
≤12h	2 (5)	24 (70.59)	
>12h	38 (95)	10 (29.41)	

Univariate and multivariate regression analyses identified the type of surgery (*p* = 0.014) and preoperative fasting time (*p* < 0.001) as risk factors for postoperative hemorrhage. Both the type of surgery (*p* = 0.050) and preoperative fasting time (*p* < 0.001) were also found to be independent risk factors for postoperative hemorrhage in partial nephrectomy (Table 4).

**Table 4:** Univariate analysis and multivariate analysis of bleeding in patients with partial nephrectomy.

Characteristic	Univariate analysis		Multivariate analysis	
	HR (95% CI)	*P*-value	HR (95% CI)	*p*-value
Hypertension	1.150 (0.445–2.970)	0.773		
Diabetes	1.286 (0.429–3.851)	0.653		
CHD	0.374 (0.037–3.770)	0.404		
BMI	0.950 (0.831–1.087)	0.455		
Age	1.001 (0.965–1.037)	0.973		
Gender	2.241 (0.876–5.734)	0.092		
Type of surgery	0.252 (0.084–0.761)	0.014	(0.237 0.056–0.999)	0.050
Tumor size	1.137 (0.865–1.495)	0.357		
RENAL score	1.111 (0.823–1.498)	0.492		
Warm ischemia time	0.955 (0.844–1.081)	0.469		
Admission heart rate	0.926 (0.870–1.987)	0.118		
Admission systolic blood pressure	0.987 (0.957–1.019)	0.428		
Admission diastolic blood pressure	0.975 (0.934–1.017)	0.24		
Preoperative creatinine	1.040 (0.007–1.074)	0.117		
Preoperative blood sodium	0.932 (0.742–1.171)	0.546		
Preoperative blood potassium	1.330 (0.338–5.236)	0.683		
Preoperative blood calcium	7.865 (0.082–753.481)	0.376		
Preoperative blood phosphate	6.036 (0.425–85.803)	0.184		
Preoperative blood magnesium	3.769 (0.003–5,340.007)	0.72		
Intraoperative heart rate	0.973 (0.927–1.021)	0.268		
Intraoperative systolic blood pressure	0.981 (0.954–1.008)	0.167		
Intraoperative diastolic blood pressure	0.979 (0.935–1.025)	0.363		
Surgery duration	0.999 (0.992–1.007)	0.895		
Intraoperative blood loss	0.998 (0.994–1.003)	0.532		
Fasting time	45.600 (9.189–226.284)	<0.001	(46.773 8.982–243.556)	<0.001

## 5 Discussion

Renal cancer accounts for more than 3% of all new cancer cases worldwide, and its incidence is rising annually (13, 14). Numerous studies have shown a threefold increase in the number of partial nephrectomies performed annually, while cases of radical nephrectomy have decreased by 40% in recent years. We found that radical nephrectomy increases the risk of surgical wound infection by 195%, sepsis by 160%, pancreatitis by 110%, blood transfusion by 100%, acute thromboembolism by 90%, acute respiratory failure and acute kidney disease by 60%, intestinal obstruction by 40%, and intensive care unit admission by 20% (15). Consequently, partial nephrectomy is becoming increasingly common in clinical practice. This study compares the effects of short-term and long-term preoperative fasting in patients undergoing partial nephrectomy and analyzes the risk factors for postoperative hemorrhage to provide clinical reference for physicians.

Previous studies have generally considered long-term preoperative fasting to be detrimental (9, 16–19). This issue is also present in partial nephrectomy, where some patients may need to fast for extended periods due to surgical scheduling, potentially causing adverse effects. Long-term preoperative fasting can lead to dehydration, electrolyte imbalance, and impaired cardiac and renal function. It also increases the body's stress response, potentially negatively affecting surgical tolerance and postoperative recovery. However, in this study, it was found that long-term preoperative fasting resulted in less loss of blood calcium, magnesium, phosphorus, and hemoglobin, differing from previous studies. This might be because long-term fasting leads to blood concentration and reduced plasma volume, thereby decreasing intraoperative blood loss due to lower intravascular pressure. Additionally, preoperative dehydration might reduce electrolyte loss. However, this study did not account for postoperative hospital stay and adverse outcomes. Gisele et al. (20) found that shortening preoperative fasting time can reduce the occurrence of postoperative discomfort and complications. Therefore, while long-term fasting may reduce the loss of ions and hemoglobin, it could adversely affect postoperative prognosis.

There are relatively few reports on prolonged fasting and water restriction before surgery, but multiple studies suggest that extended fasting may have certain benefits for lowering blood pressure, reducing cardiovascular risk, and extending lifespan (21–23). Ezpeleta et al. (21) explored the effects of prolonged fasting, noting significant benefits in weight loss and blood pressure reduction, though the long-term benefits require further study. Wilhelmi et al. (22) investigated the effects of fasting on healthy lifespan and cognitive function, highlighting that extended fasting can enhance intrinsic defense mechanisms and reduce cardiovascular risk. Sang et al. (24) studied the impact of prolonged fasting on the gut microbiota of obese individuals, finding that fasting significantly altered the composition and metabolic capacity of the gut microbiome. Reasonably timed fasting and water restriction may offer certain health benefits. During fasting, the body activates the process of autophagy, which breaks down and recycles damaged or aging cell components, helping to remove harmful substances from the body and promoting cellular renewal. Fasting also lowers blood glucose and insulin levels, which may help improve insulin sensitivity and regulate blood sugar fluctuations. However, excessive fasting and water restriction can lead to potential risks such as dehydration, electrolyte imbalances, and nutrient deficiencies. Therefore, the duration of fasting and water restriction should be managed carefully.

Hemorrhage is one of the common complications following partial nephrectomy (10, 25, 26). By comparing the control group and the hemorrhage group after partial nephrectomy, it was found that the type of surgery significantly impacts hemorrhage (*p* = 0.023). Robot-assisted partial nephrectomy had a greater impact on postoperative hemorrhage compared to laparoscopic partial nephrectomy. This finding differs from previous studies and may be related to the shorter implementation period and less mature technique of robot-assisted partial nephrectomy at our center. The comparison between the control group and the hemorrhage group also showed that preoperative fasting time significantly impacts hemorrhage (*p* < 0.001), with short-term fasting having a greater impact on hemorrhage. This could be related to blood concentration after fasting.

Analysis of risk factors for postoperative hemorrhage after partial nephrectomy identified surgical method (*p* = 0.014) and preoperative fasting time (*p* < 0.001) as significant risk factors. Both surgical method (*p* = 0.050) and preoperative fasting time (*p* < 0.001) were also found to be independent risk factors for postoperative hemorrhage. Tsai et al. (26) similarly identified preoperative fasting time and surgical duration as risk factors for postoperative hemorrhage after partial nephrectomy. Bai et al. (27) also found preoperative fasting time and surgical duration to be significant risk factors in their study. Lorenzo et al. (28) indicated that robot-assisted partial nephrectomy is superior to laparoscopic and open surgeries in reducing transfusions and overall complications, which contrasts with our findings. This discrepancy may be related to the surgeon's experience. The robotic system provides a three-dimensional high-definition view and seven degrees of freedom in surgical instruments, allowing more precise control and reducing damage to surrounding blood vessels and tissues. Despite the enhanced precision offered by robotic systems, the surgeon must still possess adequate robotic surgical experience. In our study, the higher incidence of postoperative hemorrhage in robot-assisted partial nephrectomy may reflect the single-center experience and the relatively short period since the introduction of robotic surgery at our institution.

Long-term preoperative fasting should be a greater focus for clinicians and nurses. Although our study found short-term fasting to be a risk factor for postoperative hemorrhage, most previous studies suggest that long-term fasting adversely affects patient prognosis. Therefore, the duration of preoperative fasting should be reasonably adjusted based on the patient's condition.

This study has some limitations. Firstly, the sample size is relatively small, which may introduce bias in the analysis. Additionally, as the study was conducted in a single center, the lack of multicenter data may limit the generalizability of the findings.

## Data Availability

The datasets presented in this study can be found in online repositories. The names of the repository/repositories and accession number(s) can be found in the article/Supplementary Material.
